# Lifestyle Intervention for Patients with Nonalcoholic Fatty Liver Disease: A Randomized Clinical Trial Based on the Theory of Planned Behavior

**DOI:** 10.1155/2022/3465980

**Published:** 2022-09-12

**Authors:** Narges Mobasheri, Leila Ghahremani, Ebrahim Fallahzadeh Abarghooee, Jafar Hassanzadeh

**Affiliations:** ^1^Department of Health Promotion and Education, Faculty of Health, Shiraz, Shiraz University of Medical Sciences, P.O. Box 75541-71536, Shiraz, Iran; ^2^Department of Persian Medicine, School of Medicine, Shiraz University of Medical Sciences, Shiraz, Iran; ^3^Department of Epidemiology, School of Health, Shiraz University of Medical Sciences, Shiraz, Iran

## Abstract

**Introduction:**

Nonalcoholic fatty liver disease (NAFLD) is the most common chronic liver disease, accounting for about 25% and 33% of the world's adult population and Iranians, respectively. There is currently no effective therapeutic agent available for the treatment of NAFLD. However, lifestyle modifications aimed at weight loss have been introduced as a cornerstone of NAFLD management. The aim of the present study was to evaluate the effect of educational intervention on lifestyle and anthropometric indices in patients with NAFLD.

**Methods:**

The randomized controlled clinical trial was performed on 87 overweight or obese patients with NAFLD, including intervention (*n* = 42) and control (*n* = 45) groups. The intervention received 8 training sessions based on theory of planned behavior (TPB), while the control groups received nutritional and physical activity recommendations from their internal specialist and nutritionist. Analyses were carried out based on data collected from TPB constructs and anthropometric indices (weight, body mass index (BMI), waist size, and waist-hip ratio (WHR)) at three stages (before intervention and two and 12 weeks after the intervention), as well as data obtained from liver enzymes (alanine aminotransferase (ALT) and aspartate aminotransferase (AST)), and ultrasound-based grading of NAFLD at two stages (before and 12 weeks after the intervention).

**Results:**

After the intervention, a significant difference was found between the intervention and control groups, resulting in the increased mean scores of TPB constructs, decreased liver enzymes, and decreased degree of NAFLD ultrasound. In addition, the intervention group experienced more activity and healthy diet as compared with the control group. Anthropometric indices showed only a significant decrease in BMI in the intervention group (*p* < 0.05).

**Conclusions:**

TPB-based training, as compared with traditional training, is a more effective and cost-effective strategy for altering nutritional behavior and physical activity of patients with NAFLD.

## 1. Introduction

Nonalcoholic fatty liver disease (NAFLD) is the most common chronic liver disease worldwide, showing a global prevalence of 25% with the highest prevalence rate in the Middle East (31.8%) [1]. The prevalence of NAFLD is increasing in Iran, accounting for about one-third of Iranians suffering from this disease [2]. Despite the small impact of heredity, age, and sex as risk factors on disease progression [3, 4], there are a wide variety of studies demonstrating that NAFLD has a close and two-way relationship with the components of metabolic syndrome (MetS), such as obesity, type II diabetes, and hyperlipidemia [5, 6]. Importantly, MetS components are considered as the most important risk factors for the development of NAFLD [7]. Potential consequences of NAFLD include higher mortality than the general population, deaths from cardiovascular disease [8], NAFLD-induced cirrhosis and cancer [3], increased risk of colon cancer, metabolic bone disease (MBD), and chronic kidney disease (CKD) [4], as well as impaired quality of life in patients with NAFLD [9, 10]. Therefore, NAFLD can be considered as an emerging public health issue because of its potential effects on the individual as well as its heavy economic burden on the family and society. Unfortunately, there is currently no effective therapeutic agent for the treatment of NAFLD [11]. The prevalence of NAFLD is significantly dependent on a person's lifestyle [4]. Of note, patients with nonalcoholic fatty liver (without changes in tissue and function of the liver) (NAFL) do not require medication, and lifestyle modifications with the goal of weight loss are still the basis of clinical guidelines [3, 4, 11]. Lifestyle modifications in patients with NAFLD include diet, physical activity, and weight loss [3, 4].

There are a wide variety of studies focusing on the type of diet for patients with NAFLD [12–14], among which Mediterranean diet (MD) is considered one of the most effective diets used for weight loss and decreased cardio-metabolic risks associated with NAFLD [4, 15, 16]. On the other hand, the guidelines for the management of NAFLD suggest that the optimal duration of physical activity should be 150–200 minutes per week for 3 to 5 days [3, 4], and weight loss by 7–10% improves liver enzymes and tissue changes [4]. Since NAFLD is becoming a crucial global health concern, there is a need to raise awareness about its prevention and treatment [17]. On the other hand, research experience showed that theoretical-based studies provide more favorable results for planning, implementing, and evaluating educational and research interventions [18]. The aim of this study was to first understand and then change the physical activity and nutrition behaviors in NAFLD patients with the help of NAFLD guidelines [1, 4] and theory of planned behavior (TPB), one of the behavior change models in health education. Some studies confirmed the effectiveness of TPB in understanding and modifying physical activity and healthy eating behavior [19–21]. However, there is no study on lifestyle changes in patients with NAFLD using health education based on behavioral models. On the other hand, studies not only introduce TPB as a suitable model to explain lifestyle intentions and behaviors but also consider it as an effective model for improving the lifestyle of patients with diabetes, obesity, and cardiovascular disease [22–24]. TPB suggests that the main determinant of behavior is the intention of an individual to be involved in behavior and perceived control over behavior [25]. Intention is influenced by three factors, namely, attitude (the degree of desirability or undesirability of a behavior from an individual's point of view), subjective norm (the effect of social pressure perceived by an individual to do or not to do a certain behavior), and perceived behavioral control (PBC, the degree to which a person feels about doing or not doing a behavior is under the control of his/her will) [26].

Based on the above, the purpose of the present study was to use TPB to change the physical activity and nutrition behaviors of patients with NAFLD.

## 2. Material and Methods

### 2.1. Ethics Statement

The present study was conducted in accordance with the guidelines of the Helsinki Declaration, IR.SUMS.REC.1398.086. The research protocol was approved by Shiraz University of Medical Sciences. Written informed consent was obtained from all patients.

### 2.2. Study Design

The present study was a two-arm parallel randomized controlled clinical trial performed on 87 overweight and obese NAFLD individuals confirmed by ultrasound between December 15, 2019, and March 19, 2020. The sample size calculation was performed using PASS software version 15 [27]. A sample size of 40 for each group achieves 90% power and an effect size of 0.504 to detect a difference of means in the research of Shafieinia et al. with a mean score of 18.3 in subjective norms, with an estimated standard deviation of 3.4, and with a significance level (alpha) of 0.05 using a two-sided paired-sample *t*-test. With a 10% dropout, the final sample size was considered to be 50 students for each group [20].

Potential participants were eligible for the study if they met the following inclusion criteria: age 20–50 years old with minimum literacy, being in grades 1 and 2 fatty liver, access and ability to use a mobile phone, no alcohol consumption, no pregnancy and breastfeeding, no use of liver medications such as calcium channel blocker and high doses of artificial estrogens, no history of hypothyroidism and Cushing's syndrome, no Ray and HELLP syndrome, no renal failure and stones, and no limitation of physical activity. Patients were excluded from the study if they met one of the following exclusion criteria: more than one absence from the educational session, failure to participate in pretest or posttest, unwillingness to continue participation in the study, and immigration.

### 2.3. Patients

**Figure 1:**
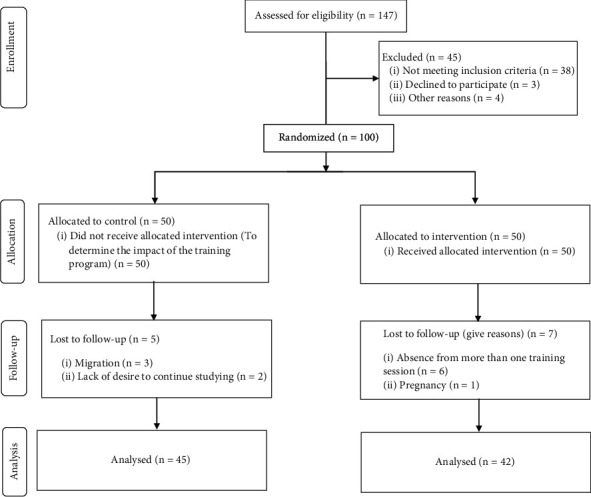
Summary of patient flow diagram.

Three internal specialists affiliated with Imam Reza Hospital (the only public hospital in Lar, Fars province, Iran) diagnosed NAFLD patients. The qualified patients (87) were included in the study based on the abovementioned inclusion criteria. Participants were selected through stratified random sampling considering four strata of gender (male or female), age groups (less than and over 35 years old), body mass index (BMI; less than and over 30), and fatty liver grades (grades 1 and 2). Random allocation was done by computer-generated random numbers. Afterwards, the patients were divided into two groups, namely, educational intervention groups (Figure 1). Participants in the intervention group received 8 group educational sessions (480 minutes in total) and received weekly reminders (one message per week) by WhatsApp or SMS messengers to change and maintain their behavior (Supplementary table 1), while the control group only received the usual recommendations given by their physician about illness, nutrition, and physical activity with no educational intervention. At the end of the trial (12 weeks after the intervention), the educational materials used in the intervention group were presented to the control group in the form of an educational booklet and a face-to-face educational session. Patients were asked to inform the researcher if they took herbal and chemical supplements related to fatty liver disease.

### 2.4. Study Tools and Evaluations

We collected demographic information and measured liver enzymes (alanine aminotransferase (ALT), aspartate aminotransferase (AST)) (Saheb Al-Zaman Clinic in Lar). In addition, fatty changes were measured in the liver by ultrasound at Imam Reza Hospital. It is worth noting that all of the measurements were carried out in two phases (before and end of the trial). TPB constructs and anthropometric indicators (weight and BMI determined as weight in kg divided by height in meters squared, waist circumference quantified at the minimum circumference between the iliac crest and the last rib, and the ratio of waist circumference to hip circumference (WHR) determined as the waist in cm divided by hip circumference at the maximum protuberance of the buttocks in cm) were measured in three phases (before the intervention and two and 12 weeks after the intervention) by the researcher using a questionnaire, a stadiometer, a meter, and a fixed balance.

All assessments were performed simultaneously in the morning, and participants were fasted.

### 2.5. TPB Constructs

TPB comprises five main constructs, namely, attitude, subjective norm, PBC, intention, and behavior. The TBP-based guideline related to the questionnaire design [28] was used to design physical activity and healthy nutrition questionnaires. Two, 4, 2, and 2 questions were designed for attitude, subjective norm, PBC, and intention constructs of the physical activity, respectively. Furthermore, the normalized International Physical Activity Questionnaire (IPAQ) (Short Form) [29, 30] was used to measure the behavior construct of physical activity. Eleven, 11, 10, and 10 questions were designed for attitude, subjective norm, PBC, and intention constructs of the healthy nutrition, respectively. In addition, the normalized 14-item MD Adherence Screener (MEDAS) questionnaire was used to measure the behavior construct of healthy nutrition [31]. Participants responded to the researcher-made questionnaire according to Likert on a 5-point scale. The validity of the researcher-made questionnaire was evaluated by ten health education specialists. The content validity ratio (CVR) for physical activity and healthy nutrition were 0.90 and 0.85, respectively. Additionally, the content validity index (CVI) was 0.90 and 0.90 for physical activity and healthy nutrition, respectively. For validity assessment, the questionnaire was completed by 100 NAFLD patients, and Cronbach's alpha coefficients for each construct were estimated from TPB. Attitude for physical activity and healthy nutrition was 0.79 and 0.81, respectively; subjective norm for physical activity and healthy nutrition was 0.81 and 80, respectively; PBC for physical activity and healthy nutrition was 0.80 and 0.89, respectively; and intention for physical activity and healthy nutrition was 0.78 and 0.85, respectively.

### 2.6. Educational Sessions in the Intervention Group

The intervention group received education in 8 sessions (four 2-hour sessions with 15 minutes' rest between each hour) relying on the change of intention and behavior of physical activity and healthy nutrition. Educational interventions for each TPB construct were provided using the Behavior Change Technique (BCT) Taxonomy [32]. Educational content included the MEDAS guide and chapter 1 of the Prevention of Cardiovascular Disease through MD [33] for healthy nutrition behavior. Additionally, chapter 4 of the American Physical Activity Guide (Second Edition) was used for physical activity [34]. To change and reinforce the behavior, participants were given a raw form containing a weekly physical activity progress chart at the end of each session. Furthermore, participants were assessed nutritionally at the beginning of each session using the MEDAS questionnaire. For this purpose, the rate of changes in the physical activity and nutrition behaviors were immediately measured before each educational session.

Moreover, experiences of successful people in changing behaviors were shared with the participants in each educational session. In order to maintain the behavior for 12 weeks, the intervention was sent by WhatsApp or SMS text messaging, motivational sentences and messages, and posters related to physical activity and healthy nutrition. Descriptions of the training sessions and the training methods and techniques used by BCT are provided in Supplementary table 1.

### 2.7. Statistical Analysis

The normality of data was measured by the Shapiro-Wilk test. The repeated measures test was performed to investigate the effect of education on TPB constructs and anthropometric indicators, such as weight, BMI, waist circumference, and WHR. In this analysis, education was used as a subject factor, and three time points (baseline and two and 12 weeks after the intervention) were used as a within-subject factor. Within-subject and between-subject comparisons of enzymatic changes in the liver (ALT and AST) were measured by Mann-Whitney *U* and Wilcoxon tests. Moreover, sign and Chi-square tests were conducted to compare changes in the degree of NAFLD ultrasound. All statistical analyses were performed by SPSS software version 26.

## 3. Results

Demographic characteristics in the intervention and control groups showed no significant differences (Table 1). In addition, no significant differences were found in the changes in liver enzymes, degree of NAFLD ultrasound, anthropometric indicators, and TPB constructs at the beginning of the study in both groups. Importantly, there was a significant difference in TPB constructs, physical activity, and nutrition behaviors between the intervention and control groups after education. Especially, the mean scores in the intervention group increased dramatically in time points 2 and 3 (for example, two and 12 weeks after the intervention), as illustrated in Tables 2 and 3.

The weight, waist circumference, and WHR variables substantially varied during the three time periods (*p* < 0.001), but there was no significant difference between the intervention and control groups (*p*_wight,waist,WHR_ = 0.843, 0.605, and 0.281). BMI also showed similar behavior to weight, waist, and WHR, except that in the third time point (12 weeks after the intervention). Results from BMI in the intervention group exhibited a significant decrease, as compared with the control group (*p* = 0.021) (Table 4).

Table 5 shows changes in the physical activity and adherence to MD in a categorized manner. Our findings showed that patients in the intervention group had more physical activities and better adherence to MD after the intervention, in which these changes were significant. Results from the present study showed that none of the participants in the control group was able to achieve the goal of losing 7% to 10% of baseline; however, 26% of the participants in the intervention group achieved this goal. This difference between the groups was also statistically significant (*p* < 0.001) (Table 5).

The means of enzymatic changes in the liver (ALT and AST) in the educational group were 31.57 and 17.12 after the intervention, respectively, showing a significant difference (*p* < 0.001). However, the control group exhibited no statistically significant difference in the enzymatic changes between the beginning and end of the study (*p*_ALT and AST_ = 0.672 and 0.194) (Table 6). At the end of the intervention, a significant difference was found in ultrasound changes of NAFLD grade in the intervention group (*p* = 0.002) and between the intervention and control groups (*p* = 0.009) (Table 6).

## 4. Discussion

The present study evaluated the lifestyle change of obese and overweight NAFLD patients using 480 minutes of TPB-based training. In addition, anthropometric, laboratory, and ultrasound changes were examined to prove the theoretical findings.

Attitude, subjective norm, PBC, intention, and behavior are the main constructs of TPB. In the present study, an attempt was made to improve the scores of the model constructs using the existing guidelines on TPB-based training [35]. The weaknesses and strengths of the participants were first detected by identifying the current situation, and then, the educational intervention was performed based on the information obtained from the first step. Similar studies were performed on changing the physical activity and nutrition behaviors to achieve success or failure in lifestyle changes [36–39]. For example, a study conducted by Parrott et al. could not change the attitude of the participants [38], because the communication channel used for training was email. However, there is evidence emphasizing face-to-face educational activities and engaging people with the subject as one of the effective ways to change attitudes [40]. In addition, a study carried out by Sanaeinasab et al. was unable to change the subjective norm of its audience because of the fact that only participants in training sessions were involved during the training [39]. In contrast, in the present study, the families of patients with NAFLD were participated in the training sessions, who were considered as suitable companions and incentives to change the lifestyle of the patients with NAFLD. Undoubtedly, the type of educational intervention, the communication channel, and the study population play an important role in the final outcome.

Despite the success in changing the scores of the model constructs, the comparison of two weeks and 12 weeks after the intervention showed that the longer the time of the educational intervention, the lower the average TPB scores, indicating that the effect of the educational intervention decreased over time. Therefore, it seems that there is a need for using other models of behavior changes to promote healthy lifestyle in the long term. In a study conducted in 2014, Wasserkampf et al. compared two models TPB and self-determination theory (SDT) and demonstrated that TPB is a suitable model for behavior change. However, they proposed to use other models of health education for stabilizing behavior [41].

Results from the present study showed that training sessions let participants experience increased physical activity and use a diet with high nutrient value, resulting in decreased anthropometric indices in the intervention group. Although there is no significant difference in a great number of these changes, the weight loss as much as 7% to 10% of baseline is an important factor in NAFLD management [3]. Most importantly, 26% of the participants in the intervention group showed weight loss, demonstrating a statistically significant difference. On the other hand, guidelines for NAFLD management suggest that sudden weight loss leads to liver resistance to recovery [3, 4]. Therefore, the participants in the intervention group were asked to gradually lose weight (maximum loss of 3 kg per month) during the training interventions. At the end of the study, a significant difference was found in BMI in the intervention group, when compared with that in the control group.

Studies performed in nutrition and physical activity demonstrated that changes in dietary compositions and physical activity rates lead to improved clinical features, namely, liver enzymes and ultrasound changes in NAFLD [42–45]. Results from the present study showed that educational sessions could improve the clinical conditions of patients with NAFLD by affecting their lifestyle.

## 5. Limitations

Various studies were performed to demonstrate the effects of specific diet and physical activity on improving NAFLD [42–45], all of which addressed the desired intervention within the study period. Accordingly, it was cost-effective in one educational-supportive study, as the strength of the study.

However, this study suffered from some limitations, including that the common diet of people living in Lar city is bread and rice which have a lot of calories. On the other hand, the NAFLD management guidelines emphasize the limitations of calorie intake [1, 4]. Furthermore, less consumption of bread and rice was emphasized in educational sessions. Unfortunately, the items used in the MEDAS questionnaire do not measure bread and rice consumption. In addition, other limitations of the study included Rial (Iranian currency) fluctuations, and the rise of some food prices, such as olive oil, olives, and fish meat. Despite the willingness of participants to consume these foods, most participants were unable to purchase or consume them according to the MD guidelines. Golovaty et al.'s study conducted in 2020 showed that food insecurity might be associated independently with NAFLD and advanced fibrosis among low-income adults in the United States [46]. Therefore, future studies should pay attention to food safety and access to high-quality food. Because the present study was conducted only in one city of the province, the results could not be generalized to other cities.

## 6. Conclusion

Comparison of the intervention and control groups of the study showed that TPB-based education, in addition to making effective theoretical changes, was able to decrease BMI, improve enzymatic changes in the liver (ALT and AST), and enhance the degree of NAFLD ultrasound.

## Figures and Tables

**Table 1:** General characteristics of study participants.

		Control (*n* = 45)	Intervention (*n* = 42)	^a^ *p*
Gender (%)	Male	15 (33.33)	15 (35.71)	0.826
	Female	30 (66.67)	27 (64.29)	

Level of education (%)	Under the diploma	12 (26.7)	8 (19.0)	0.690
	High school diploma	22 (48.9)	22 (52.4)	
	Bachelor	11 (24.4)	12 (28.6)	0.563^b^

Age (years)		36.44 ± 8.414	35.43 ± 7.881	

Data are means ± SDs. ^a^Obtained from Chi-square test. ^b^Obtained from independent *t*-test.

**Table 2:** Mean and standard deviation of TPB constructs for healthy nutrition over three time periods.

Constructs	Group	Time			^a^ *p*		
		Baseline	Two weeks after intervention	12 weeks after intervention	Time	Group	Time × group
Attitude toward healthy nutrition	Control group (*n* = 45)	27.29 ± 0.63	28.64 ± 0.69	28.19 ± 0.71	<0.001	<0.001	<0.001
	Intervention group (*n* = 42)	29.07 ± 0.73	70.90 ± 0.50	39.93 ± 0.16			
^a^ *p*		0.069	<0.001	<0.001			

Subjective Norme toward healthy nutrition	Control group (*n* = 45)	30.0 ± 0.96	31.36 ± 0.72	31.33 ± 0.73	<0.001	<0.001	<0.001
	Intervention group (*n* = 42)	30.45 ± 0.88	40.95 ± 0.61	39.81 ± 0.76			
^a^ *p*		0.686	<0.001	<0.001			

Perceived behavioral control (PBC) toward healthy nutrition	Control group (*n* = 45)	30.78 ± 0.73	31.40 ± 0.63	31.29 ± 0.74	<0.001	<0.001	<0.001
	Intervention group (*n* = 42)	30.57 ± 0.95	31.04 ± 0.67	37.10 ± 0.80			
^a^ *p*		0.863	<0.001	<0.001			

Intention toward healthy nutrition	Control group (*n* = 45)	31.13 ± 0.72	31.84 ± 0.74	31.78 ± 0.75	<0.001	<0.001	<0.001
	Intervention group (*n* = 42)	32.79 ± 1.06	40.86 ± 0.72	39.81 ± 0.86			
^a^ *p*		0.198	<0.001	<0.001			

Healthy nutrition behavior	Control group (*n* = 45)	5.02 ± 0.27	5.37 ± 0.25	5.31 ± 0.30	<0.001	<0.001	<0.001
	Intervention group (*n* = 42)	5.47 ± 0.28	9.47 ± 0.26	8.26 ± 0.32			
^a^ *p*		0.246	<0.001	<0.001			

Data are means ± SDs. ^a^*p* values represent the time × group interaction (computed by analysis of repeated measures).

**Table 3:** Mean and standard deviation of TPB constructs for physical activity over three time periods.

Constructs	Group	Time			^a^ *p*		
		Baseline	Two weeks after intervention	12 weeks after intervention	Time	Group	Time × group
Attitude toward physical activity	Control group (*n* = 45)	10.49 ± 0.64	12.44 ± 0.76	11.78 ± 0.75	<0.001	<0.001	<0.001
	Intervention group (*n* = 42)	10.38 ± 60	21.05 ± 0.64	19.26 ± 0.87			
^a^ *p*		0.904	<0.001	<0.001			

Subjective norms toward physical activity	Control group (*n* = 45)	11.53 ± 0.76	12.80 ± 0.84	12.44 ± 0.86	<0.001	<0.001	<0.001
	Intervention group (*n* = 42)	10.64 ± 0.76	18.95 ± 0.81	17.86 ± 0.92			
^a^ *p*		0.390	<0.001	<0.001			

Perceived behavioral control (PBC) toward physical activity	Control group (*n* = 45)	9.04 ± 1.19	10.31 ± 1.41	9.89 ± 1.36	<0.001	<0.001	<0.001
	Intervention group (*n* = 42)	9.31 ± 1.13	26.02 ± 10.31	22.74 ± 2.26			
^a^ *p*		0.872	<0.001	<0.001			

Intention toward physical activity	Control group (*n* = 45)	4.16 ± 0.26	4.33 ± 0.29	4.20 ± 0.27	<0.001	<0.001	<0.001
	Intervention group (*n* = 42)	1.21 ± 0.28	7.19 ± 0.28	6.05 ± 0.42			
^a^ *p*		0.881	<0.001	<0.001			

Physical activity behavior (met/cal/week)	Control group (*n* = 45)	105.289 ± 27.53	189.82 ± 82.20	112.08 ± 89.50	<0.001	<0.001	<0.001
	Intervention group (*n* = 42)	137.92 ± 28.50	1035.35 ± 85.09	742.35 ± 92.64			
^a^ *p*		0.249	<0.001	<0.001			

Data are means ± SDs. ^a^*p* values represent the time × group interaction (computed by analysis of repeated measures).

**Table 4:** Mean and standard deviation of anthropometric indices over three time periods.

Constructs	Group	Time			^a^ *p*		
		Baseline	Two weeks after intervention	12 weeks after intervention	Time	Group	Time × group
Weight (kg)	Control group (*n* = 45)	77.75 ± 7.56	76.887 ± 7.473	77.64 ± 7.83	<0.001	0.843	<0.001
	Intervention group (*n* = 42)	78.58 ± 8.65	76.933 ± 8.444	75.74 ± 8.30			
^a^ *p*		0.635	0.978	0.277			

BMI (kg/m^2^)	Control group (*n* = 45)	28.98 ± 1.72	28.66 ± 1.75	28.94 ± 1.84	<0.001	0.281	<0.001
	Intervention group (*n* = 42)	28.99 ± 1.79	28.39 ± 1.80	27.96 ± 2.01			
^a^ *p*		0.986	0.470	0.021			

Waist circumference (cm)	Control group (*n* = 45)	97.01 ± 4.50	96.66 ± 4.50	97.11 ± 4.68	0.001	0.605	0.387
	Intervention group (*n* = 42)	97.56 ± 5.38	96.26 ± 4.72	95.45 ± 4.58			
^a^ *p*		0.607	0.680	0.094			

WHR	Control group (*n* = 45)	0.54 ± 0.02	0.53 ± 0.02	0.54 ± 0.02	<0.001	0.281	<0.001
	Intervention group (*n* = 42)	0.54 ± 0.02	0.53 ± 0.02	0.53 ± 0.02			
^a^ *p*		0.788	0.522	0.080			

Data are means ± SDs. BMI: body mass index; WHR: waist-hip ratio. ^a^*p* values represent the time × group interaction (computed by analysis of repeated measures).

**Table 5:** Changes of liver enzymes, adherence to diet, physical activity, and weight loss.

Classification		Control group (*n* = 45)			Intervention group (*n* = 42)			^a^ *p*		
		Baseline	Two weeks after intervention	12 weeks after intervention	Baseline	Two weeks after intervention	12 weeks after intervention	Baseline	Two weeks after intervention	12 weeks after intervention
Classification of adherence to the MED	Weak adherence (%)	27 (60)	23 (51.1)	23 (51.1)	21 (50)	0	6 (14.3)	0.393	<0.001	<0.001
	Medium adherence (%)	18 (40)	22 (48.9)	22 (48.9)	21 (50)	19 (45.2)	20 (47.6)			
	Good adherence (%)	0	0	0	0	23 (54.8)	16 (38.1)			

Classification of participants by physical activity	Active (%)	1 (2.2)	2 (4.4)	1 (2.2)	2 (4.8)	30 (71.4)	18 (42.9)	0.608	<0.001	<0.001
	Inactive (%)	44(97.8)	43 (95.6)	44 (97.8)	40(95.2)	12 (28.6)	24 (87.1)			

Success in achieving weight loss goal	Yes (%)			0			11 (26.2)			<0.001
	No (%)			45 (100)			31 (73.8)			

Data are number (frequency). ^a^Obtained from Chi-square test.

**Table 6:** Ultrasound changes of NAFLD grade and liver enzymes at baseline and end of trial in patients.

		Control (*n* = 45)			Intervention (*n* = 42)			^b^ *p*	
		Baseline	End-of-trial	^a^ *p*	Baseline	End-of-trial	^a^ *p*	Baseline	End-of-trial
Liver enzymes	ALT	36.466 ± 9.734	36.600 ± 10.688	0.672	36.452 ± 10.231	31.571 ± 11.495	<0.001	0.855	0.005
	AST	24.422 ± 8.661	23.955 ± 8.702	0.194	25.381 ± 9.057	17.119 ± 9.239	<0.001	0.627	<0.001

Fatty liver (%)	No fatty liver	0	0	^c^0.999	0	8 (19)	^c^0.002	^d^0.974	^d^0.009
	Grade I	32 (71.1)	31 (68)		30 (41.4)	24 (57)			
	Grade II	13 (28.9)	14 (31)		12 (28.6)	10 (23)			

Data are means ± SDs. ALT: alanine aminotransferase; AST: aspartate aminotransferase. ^a^Obtained from Wilcoxon test. ^b^Obtained from Mann-Whitney *U* test. ^c^Obtained from sign test. ^d^Obtained from Chi-square test.

## Data Availability

The data used to support the findings of this work are available from the corresponding author upon request. Some of the data is also provided in the Supplementary Information files.

## Supplementary Materials

Supplementary Materials The supplementary table.1 is the timeline of educational and research activities of the study.
