# Supplementary material for: Lifestyle Intervention for Patients with Nonalcoholic Fatty Liver Disease: A Randomized Clinical Trial Based on the Theory of Planned Behavior
Source: Biomed Res Int. 2022 Sep 12;2022:3465980. doi: 10.1155/2022/3465980 (PMC9484896; doi:10.1155/2022/3465980)
Supplement: Supplementary Materials — The supplementary table.1 is the timeline of educational and research activities of the study. [file 3465980.f1.docx]

**Supplementary Table. 1**

Timeline of educational and research activities

| **Training Session** | **TPB Constructs** | **Coding based on BCT** | **Methods** | **Timespan** |
| --- | --- | --- | --- | --- |
| First and second sessions | Attitude , Subjective Norm | Shaping knowledge*: Information about antecedents (4.2) Instruction on how to perform the behavior (4.1)* Natural consequences*: Information about health consequences (5.1), Salience of consequences (5.2), Information about social and environmental consequences (5.3), Information about emotional consequences (5.6)*  Social support: *general (3.1), emotional (3.3)* Comparison of behavior: *Demonstration of the behavior (6.1)*  Repetition and substitution: *Behavioral practice/rehearsal(8.1* | question and answer, group discussion, invite important people in the life of the intervention group, Friends and family | From 4/4/2020  To 25/4/2020 |
| Third and fourth sessions | Subjective Norm, Perceived Behavioral Control (PBC) | Social support: *general (3.1), emotional (3.3)*  Self-belief: *Verbal persuasion to boost self-efficacy (15.1), Mental rehearsal of successful performance (15.2), Self-talk (15.4)*  Comparison of behavior: *Social comparison (6.2)* Goals and planning: *Problem solving(1.2)* | group discussion, invite important people in the life of the intervention group, Friends and family, brainstorming |  |
| Fifth and sixth sessions | Perceived Behavioral Control (PBC), Behavior | Self-belief: *Verbal persuasion to boost self-efficacy (15.1), Mental rehearsal of successful performance (15.2), Focus on past success (15.3), Self-talk (15.4)* Comparison of outcomes: *Pros and cons (9.2), Comparative imagining of future outcomes (9.3)* Comparison of behavior: *Social comparison (6.2)* Goals and planning: *Problem solving(1.2)* | question and answer, group discussion, brainstorming |  |
| Seventh and eighth sessions | Behavior, Conclusion | Comparison of outcomes: *Pros and cons (9.2), Comparative imagining of future outcomes (9.3)* Comparison of behavior: *Social comparison (6.2)* Goals and planning*: Problem solving(1.2), Review behavior goal(s) (1.5), Review of outcome goal(s) (1.7)* | brainstorming ,group discussion |  |
| Post test 1 | Completing the self-report questionnaire  Measurement of anthropometric indicators | | | From 9/5/2020  To 13/5/2020 |
| Recall 1 to 10 | text message ( One message per week) | | | From 16/5/2020 To 18/7/2020 |
| Post test 2 | Completing the self-report questionnaire  Measurement of anthropometric indicators  Blood test and ultrasound | | | From 19/7/2020  To 25/7/2020 |
